# ^1^H NMR-Based Metabolomics Reveals the Antitumor Mechanisms of Triptolide in BALB/c Mice Bearing CT26 Tumors

**DOI:** 10.3389/fphar.2019.01175

**Published:** 2019-10-11

**Authors:** Cheng Li, Zhongfeng Li, Tianjiao Zhang, Peihuang Wei, Nuo Li, Wei Zhang, Xia Ding, Jian Li

**Affiliations:** ^1^School of Traditional Chinese Medicine, Beijing University of Chinese Medicine, Beijing, China; ^2^Department of Chemistry, Capital Normal University, Beijing, China; ^3^Hospital, Shijiazhuang University, Shijiazhuang, China

**Keywords:** ^1^H NMR, metabolomics, triptolide, CT26 tumors, antitumor

## Abstract

Triptolide, the main active ingredient in *Tripterygium wilfordii* **Hook. f. (Celastraceae)**, has shown promising effects against a variety of tumors. However, the molecular pharmacological mechanisms explaining the action of triptolide remain unknown. In this study, the CT26 colon tumor cell line was inoculated subcutaneously into BALB/c mice, and plasma samples were subjected to ^1^H NMR metabolomics analysis. The metabolic signature identified five metabolites whose levels were lower and 15 whose levels were higher in CT26 tumor-bearing mice than in normal control mice. Triptolide treatment significantly reversed the levels of nine of these metabolites, including isoleucine, glutamine, methionine, proline, 3-hydroxybutyric acid, 2-hydroxyisovalerate, 2-hydroxyisobutyrate, and low-density lipoprotein/very low-density lipoprotein. Based on the identities of these potential biomarkers, we conclude that the antitumor mechanism of triptolide might rely on correcting perturbations in branched-chain amino acid metabolism, serine/glycine/methionine biosynthesis, and ketone bodies metabolism.

## Introduction

Colorectal cancer is a malignant tumor, characterized by high incidence and high recurrence. Despite significant improvements in colon carcinoma screening, diagnosis, and therapy, the 5-year survival rate of patients with advanced colorectal cancer remains poor (Siegel et al., 2018). While surgery, chemotherapy, and radiotherapy are still the predominant treatments for colorectal cancer, their side effects are considerable. Therefore, the development of more natural products for the treatment of colorectal cancer has become a priority.

Triptolide, a small diterpenoid epoxide extracted from *Tripterygium wilfordii* **Hook. f. (Celastraceae)**, has been found to be highly effective against a variety of cancer types including colorectal cancer (Johnson et al., 2011; Liu et al., 2012; Oliveira et al., 2015) pancreatic cancer (Phillips et al., 2007), neuroblastoma (Yan et al., 2015; Jiang et al., 2018), and prostate cancer (Zhao et al., 2016; Tamgue and Lei, 2017). Trials with minnelide, a synthetic water-soluble prodrug of triptolide, have shown some promising results on pancreatic cancer, anaplastic thyroid cancers, and prostate cancer (Banerjee and Saluja, 2015; Jacobson et al., 2015; Kumar et al., 2016; Isharwal et al., 2017). Considering the excellent anticancer activity of triptolide, it has become imperative to understand its molecular pharmacological activity.

In the last 10 years, the idea of cancer as a metabolic disorder has attracted increasing attention. This change in thinking was largely due to the increased accessibility of oncometabolites (Collins et al., 2017). So far, an increasing number of oncometabolites and cancer-associated metabolic pathways have been identified in colon cancer; they include isoleucine, tyrosine, pyruvate, histidine, threonine, phenylalanine, glutamate, taurine, lactate, phosphocholine, choline, glycerol, and mannose (Mishra and Ambs, 2015; Wishart et al., 2016). Furthermore, metabolomics provides an important tool for tracing general metabolic changes in biological processes and is particularly apt for biofluids. In particular, metabolomics approaches based on nuclear magnetic resonance (NMR), liquid chromatography mass spectrometry, or gas chromatography mass spectroscopy might provide a more detailed picture of various tumors, their possible biomarkers, and therapeutic targets.

As well known, liquid chromatography–mass spectrometry technology (LC-MS) has the advantages of rapid separation and high sensitivity compared with NMR-based technology and has become the preferred method for cell metabolomics research. 1H NMR spectroscopy is a well-established, reliable, and reproducible tool in metabolomics, which allows the detection and quantification of multiple metabolites within a single experiment (Smolinska et al., 2012; Zhang et al., 2013). As a noninvasive, nondestructive, highly discriminatory, and much more robust technique when compared to MS, it could analyze many of the core metabolites needed to construct metabolic maps (i.e., amino acids, sugars, and tricarboxylic acid cycle intermediates). Furthermore, it can be quantified directly instead of the quantification spanning six orders of magnitude specific internal standards.

In the present study, the CT26 tumor cell line was inoculated subcutaneously into BALB/c mice. Plasma samples of normal control, CT26 tumor-bearing mice, and triptolide-treated mice were collected. The metabolites were analyzed by ^1^H NMR. The plasma markers of CT26 tumor-bearing mice were identified by multivariate statistical analysis, pattern recognition technology, and metabolic pathway analysis. Finally, triptolide was given to model mice with the aim of gaining a mechanistic insight into its antitumor effects.

## Materials and Methods

### Chemicals and Materials

Triptolide (purity 99%, molecular weight 360.4) was purchased from Ze-Lang Co. Ltd. (Nanjing, China) and dissolved in sterile saline to make a stock solution. Murine CT26 cells were purchased from the tumor cell library of the Chinese Academy of Medicine (ATCC CRL-2638). Cell culture materials were purchased from Thermo Fisher Scientific (Grand Island, NY, USA).

### Animal Handling and Experimental Design

A total of 55 BALB/c mice (female, body weight 25 ± 3 g) were purchased from Beijing Vital River Company (Beijing, China; Rodent license SCXK-2012-0001). The animals were housed under controlled light (12-h light/12-h dark cycle) with the temperature of 22 ± 2°C and humidity of 50 ± 10%. All mice were allowed to adapt to their environment over a period of one week before grouping. Mice were randomly allocated into three groups as follows: 1) normal control mice (15 mice, 200 μl saline solution intraperitoneally once a day); 2) CT26 tumor-bearing mice (20 mice, 200 μl saline solution intraperitoneally once a day); and 3) triptolide-treated CT26 tumor-bearing mice (20 mice, 0.4 mg/kg triptolide intraperitoneally once a day). All intervention treatments began 12 h after the implantation of CT26 cells and lasted for weeks. After that, animals were sacrificed, and the tumor masses were removed, weighted, and measured. All animal experiments were performed in accordance with institutional guidelines and following approval by the Ethics Review Committee of Beijing University of Chinese Medicine.

### CT26 Cell Challenge and Assessment

CT26 cells were routinely cultured in RPMI-1640 medium containing 10% fetal bovine serum, 2 mM L-glutamine, and 1% penicillin/streptomycin solution. The cells were cultured under standardized conditions (5% CO_2_, 100% humidity, 37 °C) and used at 75–80% confluence.

Phosphate-buffered saline (200 μl) with CT26 cells (2 × 10^6^, viability ≥95%) were injected hypodermicly into the right forelimb of mice using an insulin syringe. The tumors were measured with callipers at 2-day intervals after tumor nodules initiation. Tumor volume was calculated as (length × width^2^)/2 (Hu et al., 2015). Percent survival was determined based on the survival curve for the primary subcutaneous tumor model.

### NMR Analysis and Data Processing

The assay was performed as described previously (Psychogios et al., 2011; Nagana Gowda et al., 2015). In brief, mouse blood samples were withdrawn into a heparinized tube and centrifuged, the plasma samples were prepared as usual, and the supernatant was used for NMR analysis. All samples were analyzed by Varian VNMRS 600 MHz NMR spectrometer, ^1^H NMR spectra were acquired by water-suppressed one-dimensional (1D) Carr-Purcell-Meiboom-Gill (CPMG) pulse sequence [RD-90°- (τ-180°-τ) n- ACQ], and free induction decays (FIDs) were collected. At the same time, standard correlation spectroscopy (COSY), total correlation spectroscopy (TOCSY), heteronuclear multiple-bond correlation spectroscopy (HMBC), and J-resolved spectra of the plasma samples were obtained.

All ^1^H NMR spectra were aligned by MestReNova 7.1.0 and analyzed using SIMCA-P 12.0 with the method described previously (Psychogios et al., 2011; Nagana Gowda et al., 2015). Principal component analysis (PCA), partial least squares discriminant analysis (PLS-DA), and orthogonal PLS-DA (OPLS-DA) were used to analyze the NMR data of the samples. The coefficient plots were generated with MATLAB scripts and color-coded based on the absolute value of coefficients (r).

### Statistical Analysis

Data are expressed as the mean ± standard deviation. An independent sample *t*-test was performed between the two groups by SPSS 20.0. *p*-value <0.05 was considered to be statistically significant. An additional diagnostic model was constructed using the marker metabolites alone and a linear discrimination analysis method.

## Results

### Tumor Inhibitory Effect of Triptolide

**Figure 1:**
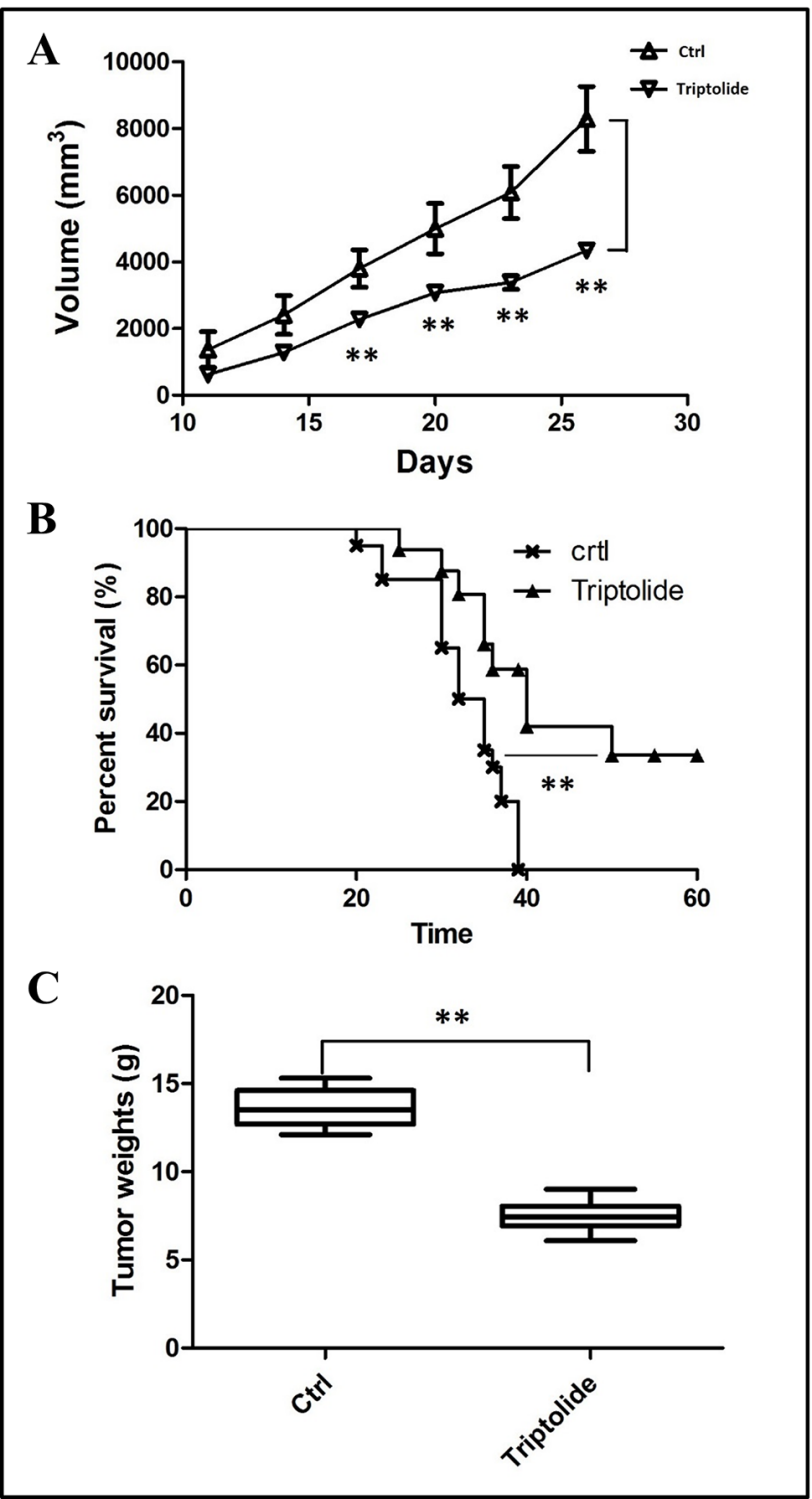
Tumor inhibitory effect of triptolide in a mouse CT26 tumor model. **(A)** tumor volumes were determined from the histograms at 5-day intervals after CT26 subcutaneous injection. **(B)** Percentage survival curves of tumor model and triptolide-treated groups. **(C)** Tumor weights (**P < 0.01, Triptolide vs. control group).

To estimate the effect of triptolide, tumor volume and weight were measured. Results showed that triptolide significantly inhibited CT26 mass growth compared with the model control group (**Figures 1A, C**). Moreover, subcutaneous CT26 tumor-bearing mice from the triptolide treatment group survived much longer than mice from the model group (CT26 alone) (**Figure 1B**). An *in vitro* evaluation of triptolide’s antitumor effect revealed that 25 and 50 nM triptolide significantly inhibited CT26 cell proliferation and migration while inducing apoptosis (data not shown, **Supplementary Figure 1**).

### ^1^H NMR Spectroscopy of Plasma

**Figure 2:**
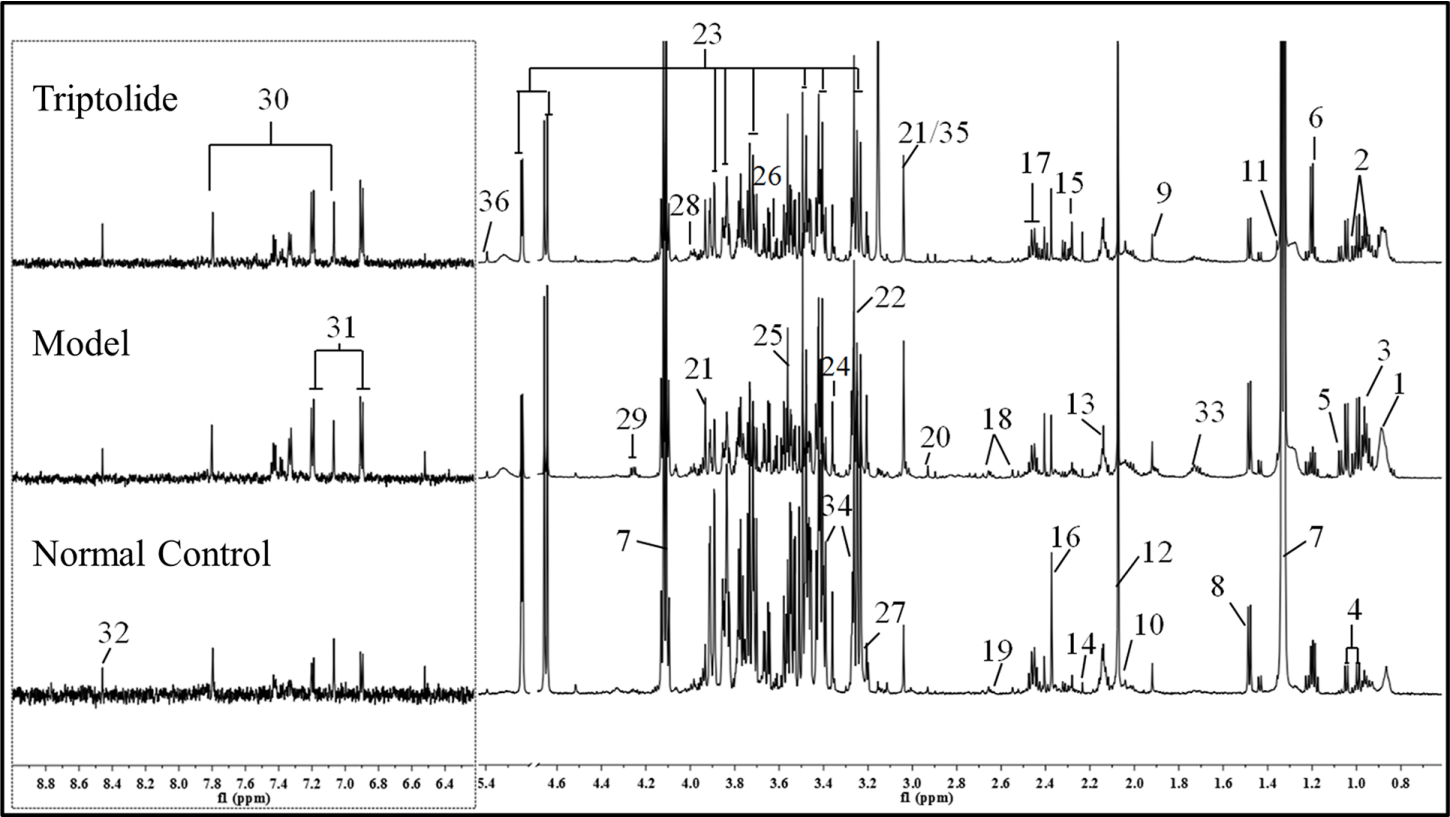
Representative 600 MHz 1H NMR spectra of plasma from normal control, model, and triptolide-treated groups. The following metabolites could be distinguished: (1) low-density lipoprotein (LDL); (2) isoleucine; (3) leucine; (4) valine; (5) isobutyrate; (6) 3-hydroxybutyrate; (7) lactate; (8) alanine; (9) acetate; (10) proline; (11) 2-hydroxyisobutyrate; (12) NAC1; (13) NAC2; (14) acetone; (15) acetoacetate; (16) pyruvate; (17) glutamine; (18) citrate; (19) methionine; (20) N,N-dimethylglycine; (21) creatine; (22) betaine; (23) glucose; (24) glycerophosphoylcholine; (25) glycine; (26) glycerol; (27) choline; (28) serine; (29) threonine; (30) methylhistidine; (31) tyrosine; (32) formate; (33) lysine; (34) taurine; (35) phosphate; and (36) allantoin.

As shown in **Figure 2**, plasma from normal control mice, CT26 tumor-bearing mice, and triptolide-treated mice were analyzed by ^1^H NMR spectra. Resonance assignments were performed according to existing literature (Psychogios et al., 2011; Nagana Gowda et al., 2015) and confirmed by COSY, TOCSY, HMBC, and J-resolved spectra. Detailed metabolite assignments were displayed in **Supplementary Tables 1** and **2** (or data not shown). A total of 24 metabolites’ levels were found to be perturbed (16 increased, 5 decreased, 2 nonremarkably, and 1 was unknown) in the plasma of CT26 tumor-bearing mice compared to normal control mice.

### Multivariate Analysis of NMR Data

**Figure 3:**
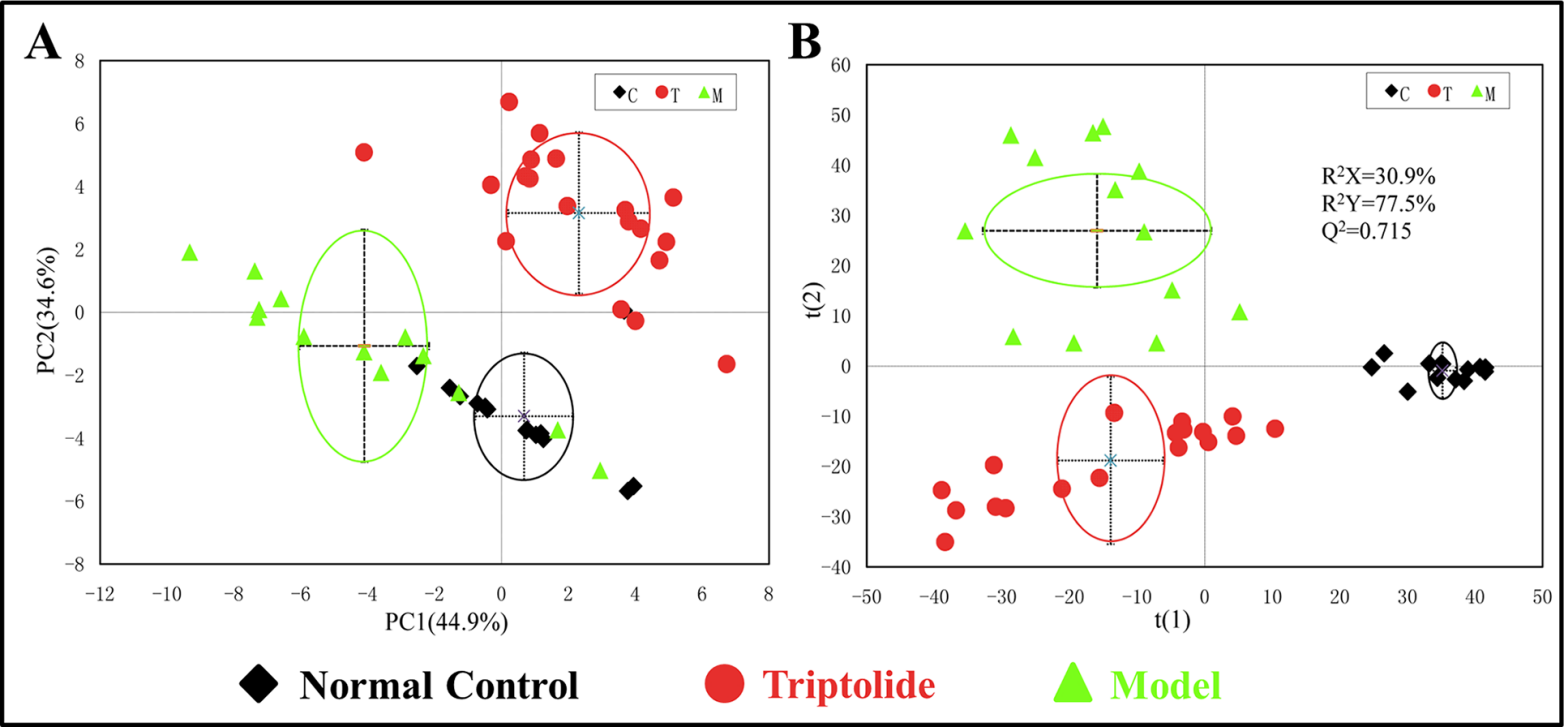
**(A)** PCA and **(B)** PLS-DA score plots of mouse plasma samples.

PCA was used to analyze the NMR data of the samples to identify the overall metabolic trend and discover possible outliers. The PCA score plots of plasma ^1^H NMR spectra displayed a significant separation between normal control, model, and triptolide-treated groups. With PC1 and PC2 values of 44.9% and 34.6%, respectively, the model could adequately discriminate between the three experimental groups (**Figure 3A**). Most of the samples were within the 95% confidence interval, and the following analysis was applied to ensure maximum information could be retrieved. The PLS-DA score plots of plasma revealed a separation between normal control, model, and triptolide-treated groups. The classification parameters of the three groups were R^2^X = 30.9% and R^2^Y = 77.5%, indicating acceptable goodness of fit and high goodness of predication (**Figure 3B**). Response permutation tests with 200 permutations showed no overfitting in the models. According to PLS-DA and validation results, the Y-intercepts of the regression lines were negative (−0.2), which indicated that the PLS-DA model was reliable for explaining and predicting the changes in the X- and Y-matrix (data not shown, **Supplementary Figure 3**).

### Identification of Metabolites

**Figure 4:**
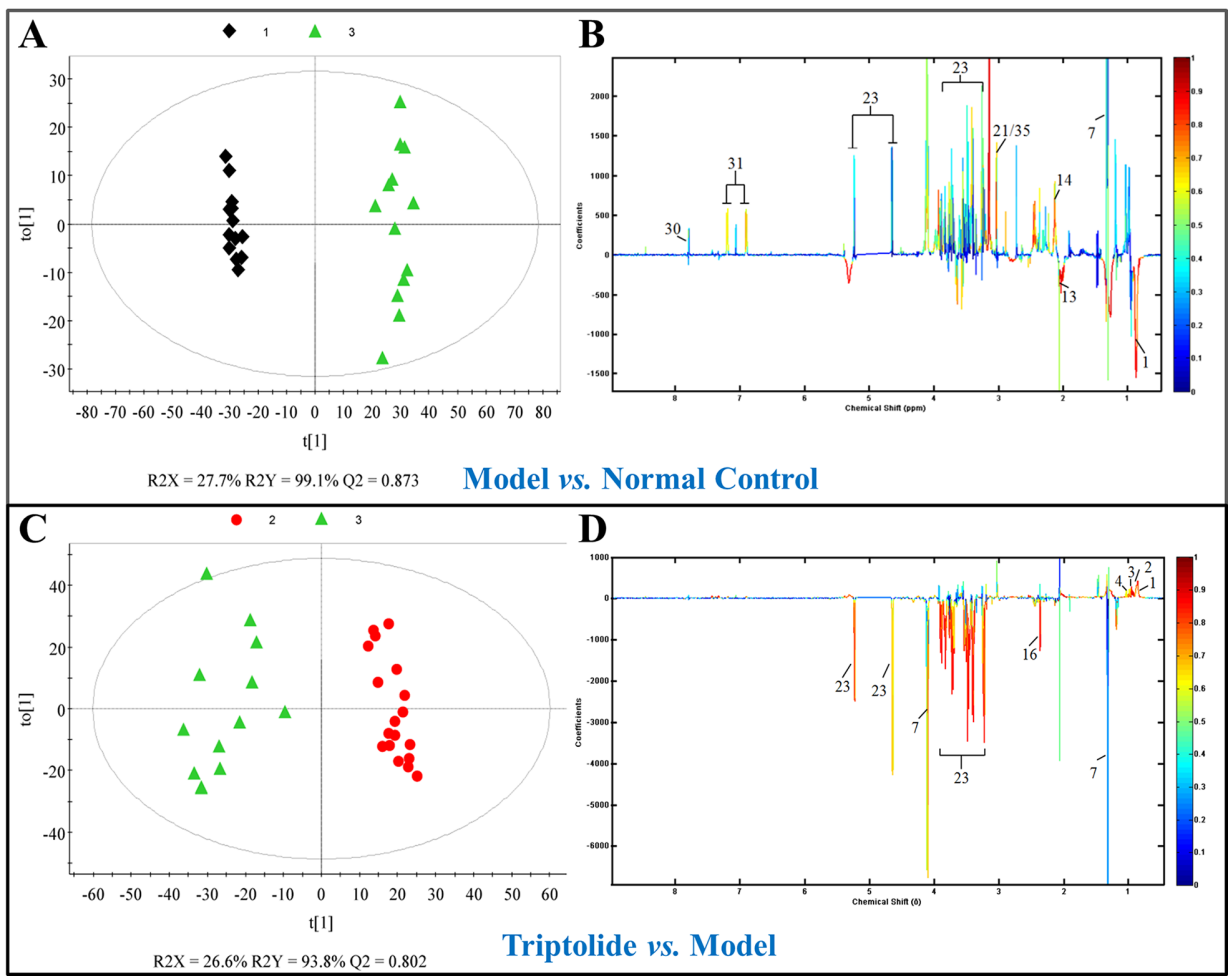
**(A**, **C)** OPLS-DA score plots and **(B**, **D)** efficient loading plots derived from the 1H NMR spectra of plasma from normal control, model, and triptolide-treated groups. Color-coding corresponds to the correlation coefficients of the metabolic variables.

**Figure 5:**
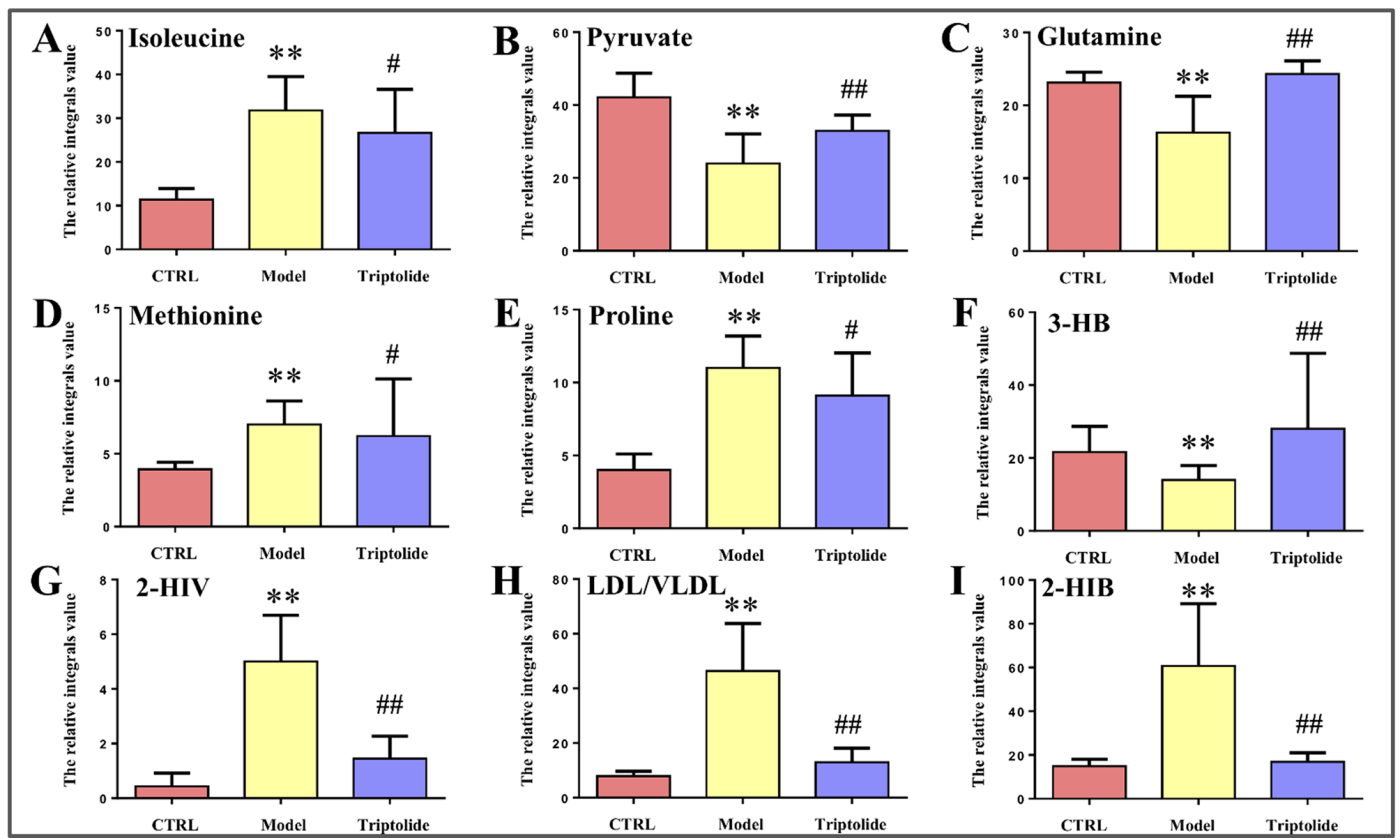
Relative integral levels of metabolites in normal control, model, and triptolide-treated groups. **(A)** Isoleucine; **(B)** Pyruvate; **(C)** Glutamine; **(D)** Methionine; **(E)** Proline; **(F)** 3-HB; **(G)** 2-HIV; **(H)** LDL/VLDL; **(I)** 2-HIB (**P < 0.01, model vs. control group; ^#^*P* < 0.05, Triptolide vs. model; ^##^*P* < 0.01, Triptolide vs. model).

As shown by the loading plots in **Figure 4**, more metabolites were increased (peaks in the positive direction) in the model group than in the normal control group (**Figures 4A, B**). This, however, was reversed by triptolide treatment, with more metabolites seen to decrease (peaks in the negative direction) in the triptolide-treated group compared with the model group (**Figures 4C, D**). According to the absolute cut-off value of the correlation coefficient (|r|) and VIP value (listed in **Supplementary Tables 1** and **2**), the metabolites with significant change (*P* < 0.05) were identified as potential biomarkers. Based on VIP > 1 and the degree of perturbation of the evaluated metabolites, the fold change of these metabolites was computed and is listed in **Table 1**. Five metabolites in the model group [glucose, pyruvate, 3-hydroxybutyric acid (3-HB), glutamine, and betaine] exhibited an obvious decrease, whereas 15 [serine, creatine phosphate, methionine, threonine, valine, allantoin, dimethylglycine, proline, isoleucine, lysine, isobutyrate, leucine, 2-hydroxyisobutyrate (2-HIB), low-density lipoprotein/very low-density lipoprotein (LDL/VLDL), and 2-hydroxyisovalerate (2-HIV)] exhibited a significant increase compared with the normal control group (*P* < 0.05 or *P* < 0.01). Following triptolide treatment, the pattern of 9 out of the 20 metabolites was significantly reversed. As **Figure 5** showed, the level of isoleucine, methionine, proline, 2-HIV, LDL/VLDL, and 2-HIB was significantly decreased, but the level of pyruvate, glutamine, and 3-HB was significantly increased compared with the model control group (*P* < 0.05 or *P* < 0.01). The above results suggest that triptolide treatment could effectively regulate the metabolic networks associated with some of these metabolites in CT26 tumor-bearing mice.

**Table 1:** Quantitative comparison of metabolites found in the plasma of normal control, model, and triptolide-treated groups.

	Metabolites	Model vs. Control		Triptolide vs. Model	
		log_2_(FC)	*p*-Value	log_2_(FC)	*p*-Value
1	Glucose	–1.00	0.001	/	/
2	Pyruvate	–0.81	0.001	0.46	0.002
3	3-Hydroxybutyrate	–0.63	0.002	1.00	0.009
4	Glutamine	–0.51	0.001	0.58	0.004
5	Beatine	–0.24	0.001	/	/
6	Serine	0.42	0.006	0.61	0.001
7	Creatine phosphate	0.62	0.002	0.44	0.001
8	Methionine	0.83	0.001	−0.17	0.036
9	Threonine	1.10	0.001	/	/
10	Valine	1.15	0.004	0.75	0.038
11	Allantoin	1.29	0.001	0.37	0.011
12	Dimethylglycine	1.43	0.001	/	/
13	Proline	1.46	0.001	−0.32	0.025
14	Isoleucine	1.48	0.001	−0.25	0.029
15	Lysine	1.53	0.001	/	/
16	Isobutyrate	1.71	0.001	/	/
17	Leucine	1.73	0.001	/	/
18	2-Hydroxyisobutyrate	2.03	0.001	−1.85	0.001
19	LDL/VLDL	2.55	0.001	−1.84	0.001
20	2-Hydroxyisovalerate	3.51	0.001	−1.78	0.001

Color-coded according to log_2_ (fold change), red mean the increased and blue mean the decreased in each group. The symbol ‘/’ showed metabolites without significant difference between the model control group and Triptolide treatment group. The color bar: 
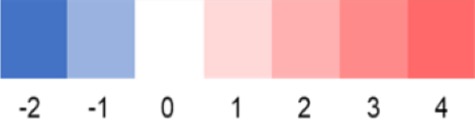
.

### Metabolic Pathway and Function Analysis

**Figure 6:**
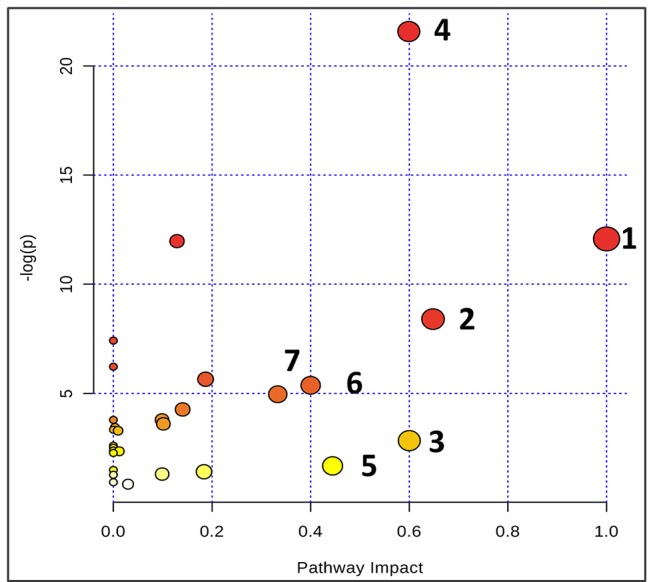
Summary of pathway analysis using Metaboanalyst. The map was gained through online analysis (http://www.metaboanalyst.ca/): (1) phenylalanine-tyrosine-tryptophan biosynthesis; (2) phenylalanine metabolism; (3) glycine-serine-threonine metabolism; (4) synthesis and degradation of ketone bodies; (5) beta-Alanine metabolism; (6) methane metabolism; and (7) valine-leucine-isoleucine biosynthesis.

Based on the identification of biomarkers, MetaboAnalyst (http://www.metaboanalyst.ca/) was used to analyze metabolic pathways to determine the involved related pathways under the present conditions. According to previous studies, pathways with an impact value >0.2 were screened out as potential targets. This yielded seven pathways related to 18 metabolites; they include 1) phenylalanine/tyrosine/tryptophan biosynthesis, 2) phenylalanine metabolism, 3) glycine/serine/threonine metabolism, 4) synthesis and degradation of ketone bodies, 5) beta-alanine metabolism, 6) methane metabolism, and 7) valine/leucine/isoleucine biosynthesis (**Figure 6**). Accordingly, these pathways might define the diagnostic perturbations characterizing CT26 tumor-bearing mice and might be the targets for triptolide treatment.

## Discussion

**Figure 7:**
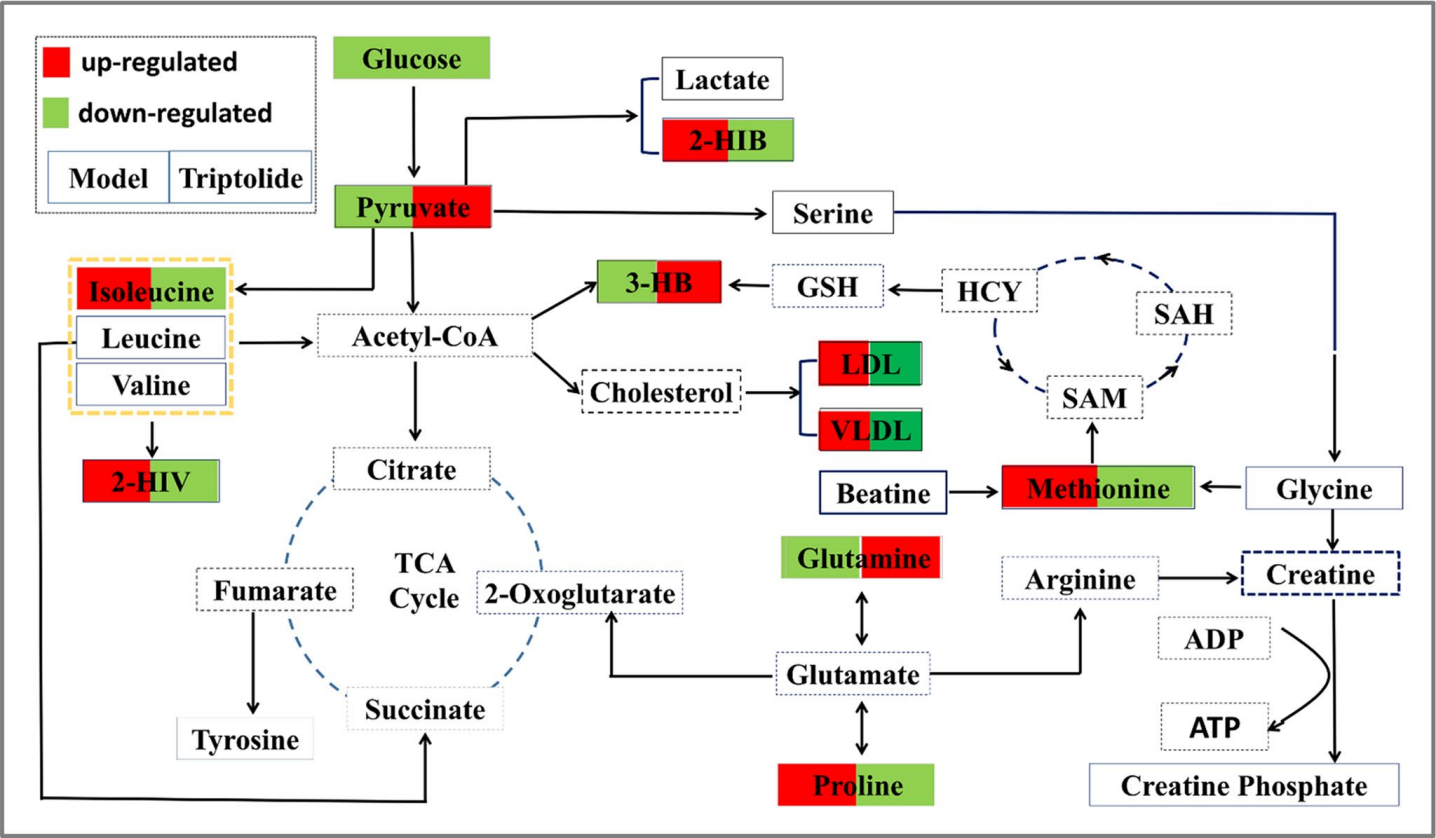
Overall profile of the metabolic network in CT26 tumor-bearing mice. The map was constructed by analyzing known metabolic pathways based on nine differentially expressed metabolites (labeled with red or green diamonds).

Growing evidence indicates that triptolide has a definite anticancer effect (Chang et al., 2007; Antonoff et al., 2009; Chen et al., 2009; Lu et al., 2011; Cheng et al., 2016; Hu et al., 2016; Reno et al., 2016; Ziaei and Halaby, 2016; Li et al., 2017). However, the compound’s molecular mechanism has not been investigated by metabolomics studies. Here, we used NMR-based metabolomics to study the *in vitro* and *in vivo* metabolic responses and the metabolic pathways of CT26 tumor-bearing mice following treatment with triptolide. Using conventional pharmacological approaches, we and others have demonstrated that triptolide indeed exerts antitumor activity (Tang et al., 2007; Liu et al., 2012; Liu et al., 2014). Furthermore, we investigated metabolic profiling of plasma and potential biomarkers of CT26 tumor-bearing mice and ways in which these changed as a result of the antitumor mechanisms of triptolide. It is worth noting that the profiling of nine metabolites could be markedly reversed, following triptolide treatment, which indicated that triptolide might help restore the normal level of metabolites in these pathways. Based on the relevant biomarkers and metabolic networks involved, we have outlined the antitumor molecular mechanisms of triptolide in **Figure 7**.

### Metabolism of Certain Amino Acids

In our study, we detected four amino acids associated with the presence of CT26 tumors and triptolide treatment: isoleucine, glutamine, methionine, and proline. These amino acids might contribute to the valine/leucine/isoleucine biosynthesis or degradation pathways and serine/glycine/methionine biosynthesis pathway in CT26 tumor-bearing mice.

Isoleucine, synthesized from threonine, is a nonpolar amino acid. The risk of colorectal adenoma has been shown to be inversely proportional to plasma levels of branched-chain amino acids; hence, their supplementation may have a beneficial effect on colorectal cancer (Budhathoki et al., 2017). However, our results differ from previously published findings. From an experimental standpoint, because of mandatory fasting during the experiment, the intake of isoleucine in the diet was likely to be zero; thus, the increase of isoleucine in plasma may be due to protein decomposition and is indicative of a negative nitrogen balance. The concentrations of amino acid in plasma could not reflect their requirements accurately, but it is nevertheless possible that branched-chain amino acid metabolism could be perturbed following triptolide treatment.

Glutamine, an inessential amino acid, plays a key role in mammalian cells and especially in cancer cells, where it is involved in cell growth, protein synthesis, and regulation of the acid balance. Under physiological conditions, glutamine is endogenously synthesized from α-ketoglutarate, *via* glutamate dehydrogenase and glutamine synthetase. In addition, glutamine and glutamate can be interconverted enzymatically and are both precursors of glutathione. Moreover, cancer cells need a large number of glutamine to grow and survive continually, which lead to extraction of cyclic glutamine acting as a “glutamine trap” (Wu et al., 2018). In the present study, a decreased level of glutamine in plasma was detected in CT26 tumor-bearing mice, whereas triptolide treatment significantly up-regulated the level of glutamine. These results suggest that triptolide might inhibit glutamine consumption in tumor cells. Several studies have reported that tumor cells are glutamine addicted. Increased glutamine consumption would manifest as a reduction of circulating glutamine (Li et al., 2017; Wang et al., 2018). Our study seems to confirm these findings and points to a valuable hypothesis for future investigation into the antitumor mechanisms of triptolide.

Methionine, an essential amino acid, is an important substrate of cysteine and taurine. Its derivative S-adenosylmethionine is a cofactor that serves mainly as a methyl donor. Methionine’s metabolic cycle, also called folate cycle, links several functional compounds, such as betaine, glycine, homocysteine, and glutathione (Kawaguchi et al., 2018). In general, compared with normal cells, cancer cells have a higher demand for methionine. Accordingly, methionine restriction could exert an antitumor effect in animal models. However, methionine is an essential amino acid that cannot be entirely removed from the diet. For example, mice fed methionine and choline deficient (MCD) diet can induce nonalcoholic steatohepatitis (Wang et al., 2014). In our study, increased methionine in the plasma of CT26 tumor-bearing mice confirms the methionine dependence of CT26 tumors. Importantly, this is the first report suggesting that triptolide might target a methionine-related metabolic pathway. As any exploration of the molecular mechanisms responsible for this interaction was beyond the scope of the present study, we believe that future work should look into the link between methionine and triptolide.

Proline, the only proteinogenic amino acid, plays a crucial part in molecular recognition, primary carbon and nitrogen metabolism, and oxidative stress and osmotic stress protection (Phang and Liu, 2012; Phang et al., 2015). Proline and glutamine are interconvertible and their metabolism is linked. Usually, proline content is relatively low. An increased proline level has been heralded as a vital variation in certain tumors (Liu et al., 2015). Our results seem to confirm these findings and further show that the increased proline level can be down-regulated by triptolide.

### Ketone Bodies Metabolism

Ketone bodies consist of, among others, 3-HB, acetone, and acetoacetate. They are produced by the liver from fatty acids during fasting or prolonged intense exercise. 3-HB is synthesized by acetyl coenzyme A in liver and serves as a source of energy by cells in the event of blood glucose is insufficient (Bonuccelli et al., 2010). Our experimental results show that the 3-HB concentration decreased significantly in the plasma of CT26 tumor-bearing mice, indicating that ketones have been used as fuel by the mitochondria of tumor cells. Based on our findings, triptolide might also inhibit the uptake of 3-HB into tumor cells.

### Other Metabolism-Related Biomarkers

Due to limitations of the MetaboAnalyst tool, biomarkers such as 2-HIV, 2-HIB, and LDL/VLDL could not be matched directly in metabolic pathway analysis. We retrieved in literature and biochemical databases to discover their relationship with colon cancer. It showed that 2-HIV was linked to branched-chain amino acid metabolism, 2-HIB and lactate were linked to pyruvate metabolism, and LDL/VLDL was involved in cholesterol metabolism. Currently, there are no reports on the correlation between these metabolites and tumors.

Overall, we report a noteworthy alteration in plasma metabolic profiling of CT26 tumor-bearing mice, and triptolide treatment reverses it significantly. The changes in metabolic profiling were characterized by an increase in plasma proline, isoleucine, methionine, 2-HIB, 2-HIV, and LDL/VLDL, as well as by a decrease in plasma pyruvate, 3-HB, and glutamine. On the basis of metabolic pathway analysis, we considered that the antitumor mechanism of triptolide relies on regulating the disorder of branched-chain amino metabolism, serine/glycine/methionine biosynthesis, and ketone bodies metabolism. In conclusion, this study can serve for potential targets of tumor drug therapy. Moreover, our research emphasizes the role of these biomarkers in cancer development and growth.

## Data Availability Statement 

All datasets generated for this study are included in the manuscript/**Supplementary Files**.

## Ethics Statement

This study was carried out in accordance with institutional guidelines, and the protocol was approved by the Ethics Review Committee of Beijing University of Chinese Medicine.

## Author Contributions

CL conceptualized the study, gave an interpretation of the data, and drafted the manuscript. ZL ran the ^1^H NMR experiments and analyzed the data. TZ and PW carried on the animal experiment and tumor cell culture. NL and WZ analyzed the data. JL designed the study and revised the manuscript. And XD designed the study and gave an interpretation of the data.

## Funding

This study was supported in part by the National Natural Science Foundation of China (NSFC) under Grants 81630080, 81873099, and 81273884.

## Conflict of Interest

The authors declare that the research was conducted in the absence of any commercial or financial relationships that could be construed as a potential conflict of interest.
