# Supplementary figures and images for: 1H NMR-Based Metabolomics Reveals the Antitumor Mechanisms of Triptolide in BALB/c Mice Bearing CT26 Tumors
Source: Front Pharmacol. 2019 Oct 11;10:1175. doi: 10.3389/fphar.2019.01175 (PMC6798008; doi:10.3389/fphar.2019.01175)

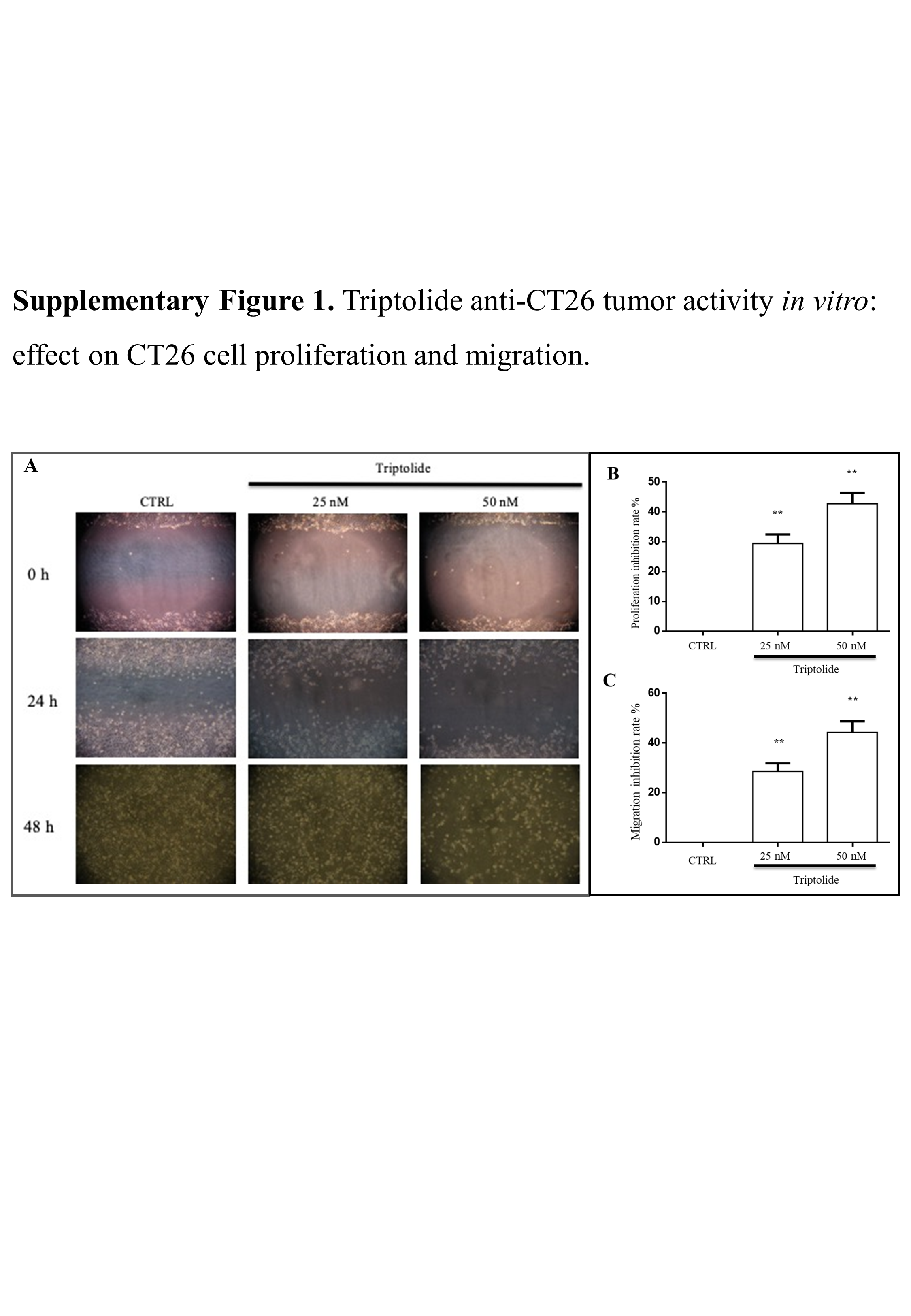

Supplement: Supplementary file 1 [file Image_1.tif]

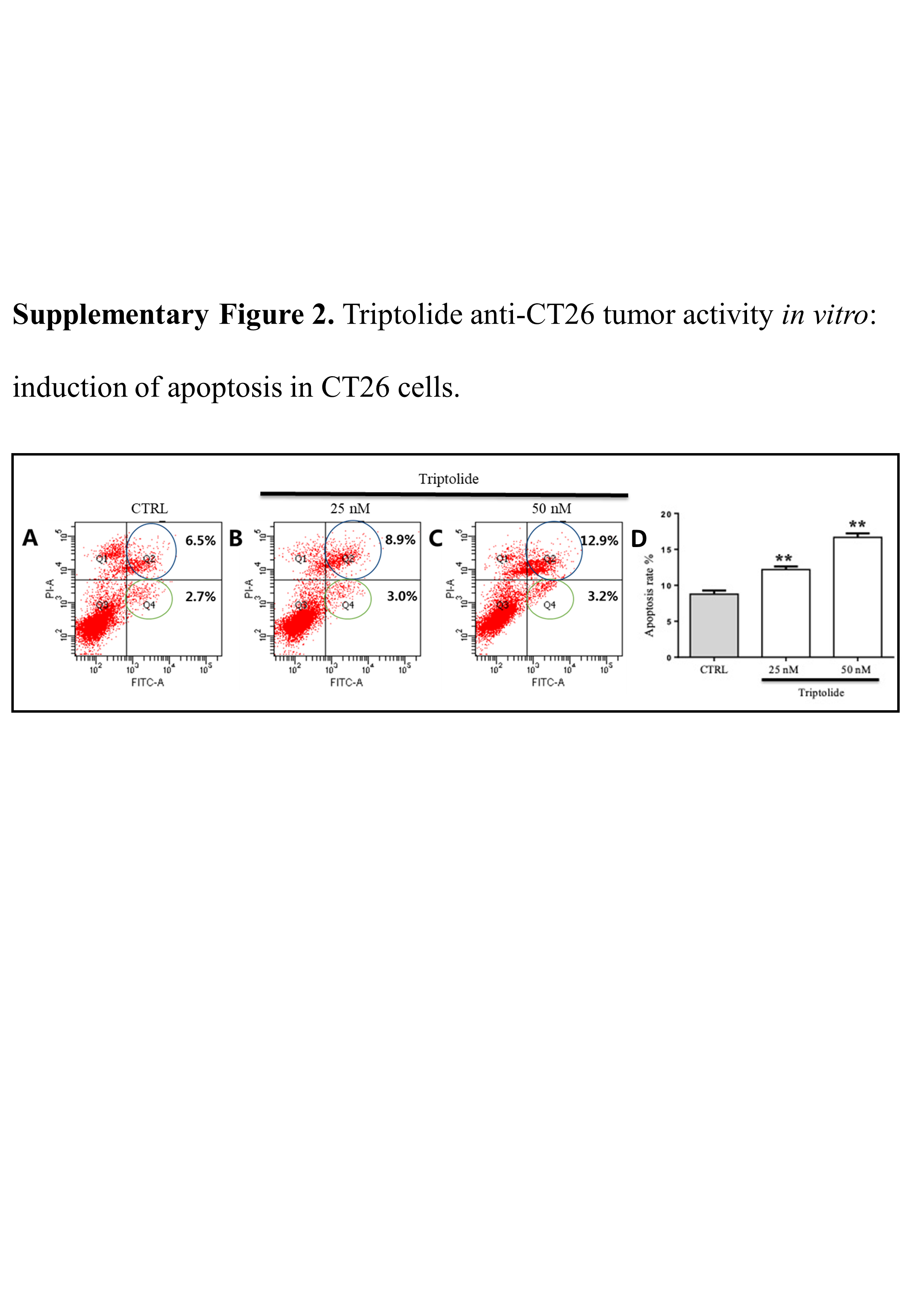

Supplement: Supplementary file 2 [file Image_2.tif]

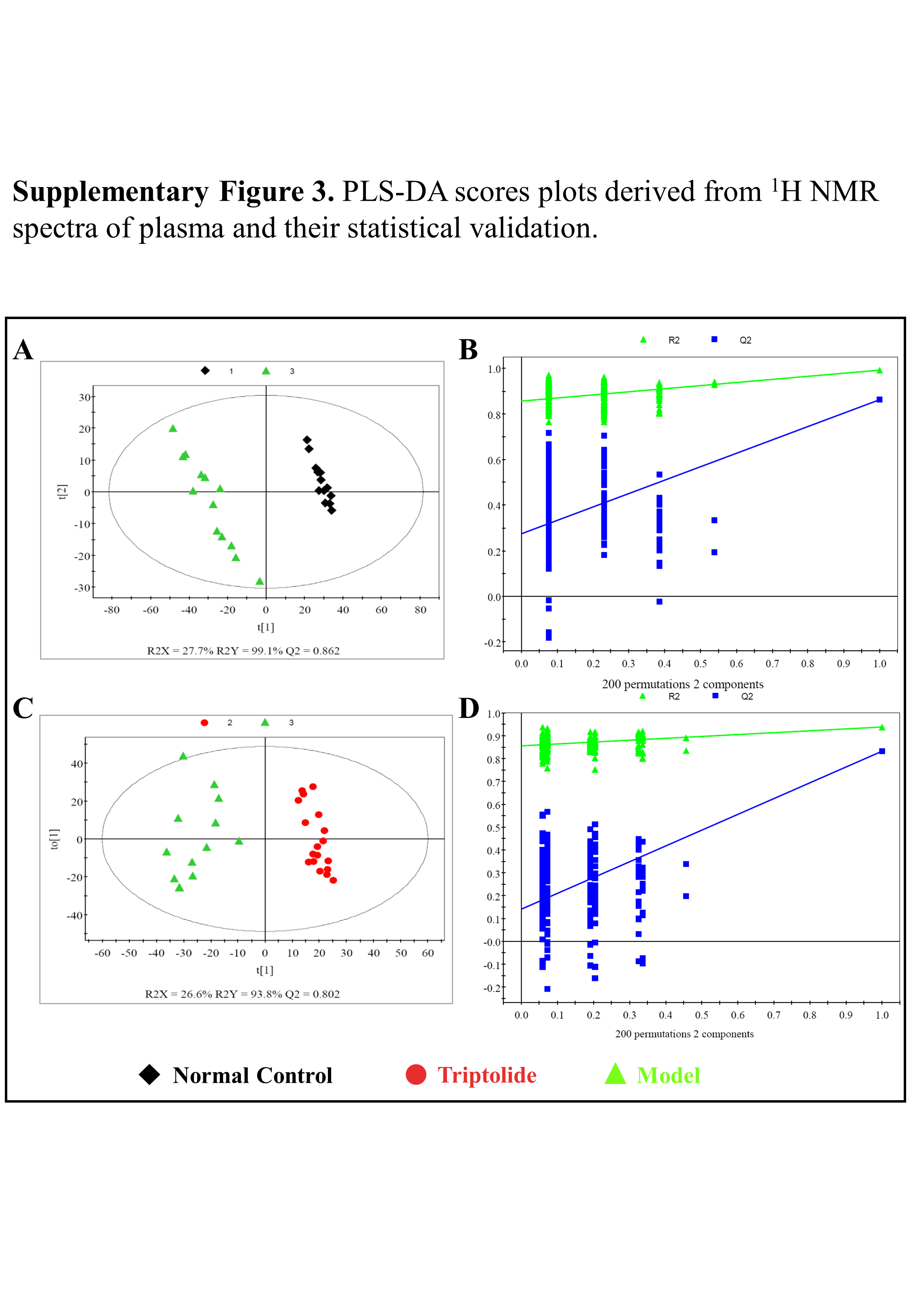

Supplement: Supplementary file 3 [file Image_3.tif]
