# Supplementary material for: 1H NMR-Based Metabolomics Reveals the Antitumor Mechanisms of Triptolide in BALB/c Mice Bearing CT26 Tumors
Source: Front Pharmacol. 2019 Oct 11;10:1175. doi: 10.3389/fphar.2019.01175 (PMC6798008; doi:10.3389/fphar.2019.01175)
Supplement: Supplementary file 4 [file Table_1.docx]

| Metabolites | ID | Chemical shift | M vs C | Interal in CTRL group ^a^  (mean ± std)×10^-2^ | Interal in Model group ^a^  (mean ± std)×10^-2^ | r ^b^ (J vs M)  (\|r\| >= 0.43) | VIP | p^c^ (kb vs chd)  (p < 0.05) |
| --- | --- | --- | --- | --- | --- | --- | --- | --- |
| 2-Hydroxyisovalerate | HMDB00407 | 0.82(d), 0.95(d) | + | 0.44 ± 0.48 | 5.0 ± 1.7 | 0.903 | 1.70972 | 0.001 |
| leucine | HMDB00687 | 0.95(m), 1.70(m) | + | 15.58 ± 4.62 | 51.53 ± 15.89 | 0.904 | 1.66797 | 0.001 |
| valine | HMDB00883 | 0.98(d), 1.03(d) | + | 15.80 ± 6.42 | 35.01 ± 19.19 | 0.693 | 1.21073 | 0.004 |
| isoleucine | HMDB00172 | 0.93(t), 0.99(d) | + | 11.40 ± 2.51 | 31.78 ± 7.73 | 0.918 | 1.71342 | 0.001 |
| LDL/VLDL |  | 0.87(m), 1.27(m) | + | 7.93 ± 1.74 | 46.34 ± 17.42 | 0.783 | 1.64286 | 0.001 |
| unknown |  | 1.43(d) | + | 4.49 ± 1.26 | 6.58 ± 1.55 | 0.654 | 1.21922 | 0.001 |
| lysine | HMDB00182 | 1.74(m), 3.04(t), 3.77(t) | + | 0.82 ± 0.52 | 2.36 ± 1.04 | 0.808 | 1.45358 | 0.001 |
| proline | HMDB00162 | 2.03(m), 2.36(m), 3.43(m) | + | 4.0 ± 1.10 | 11.0 ± 2.2 | 0.949 | 1.79948 | 0.001 |
| pyruvate | HMDB00243 | 2.36(s) | - | 42.13 ± 6.62 | 23.95 ± 8.11 | -0.814 | 1.56928 | 0.001 |
| glutamine | HMDB00641 | 2.14(m), 2.46(m), 3.79(m) | - | 23.13 ± 1.44 | 16.27 ± 4.99 | -0.661 | 1.35581 | 0.001 |
| methionine | HMDB00696 | 2.13(s), 2.63(t) | + | 3.93 ± 0.49 | 7.0 ± 1.62 | 0.790 | 1.59416 | 0.001 |
| isobutyrate | HMDB01873 | 1.05(d), 2.37(m) | + | 0.69 ± 0.80 | 2.26 ± 1.02 | 0.827 | 1.49022 | 0.001 |
| dimethylglycine | HMDB00092 | 2.93(s), 3.71(s) | + | 1.0 ± 0.47 | 2.70 ± 0.73 | 0.755 | 1.67515 | 0.001 |
| glucose | HMDB00122 | 3.24(m), 4.64(d), 5.23(d) | - | 152.74 ± 30.73 | 76.12 ± 25.19 | -0.841 | 1.63292 | 0.001 |
| creatine phosphate | HMDB01511 | 3.04(s), 3.93(s) | + | 25.34 ± 7.79 | 38.87 ± 12.35 | 0.637 | 1.30878 | 0.002 |
| 2-hydroxyisobutyrate | HMDB00729 | 1.35(s) | + | 14.88 ± 3.12 | 60.73 ± 28.47 | 0.741 | 1.49068 | 0.001 |
| 3-hydroxybutyrate | HMDB00357 | 1.19(d), 2.30(m) | - | 21.64 ± 7.02 | 13.98 ± 3.96 | -0.621 | 1.38046 | 0.002 |
| serine | HMDB00187 | 3.84(m), 3.96(m) | + | 8.74 ± 2.03 | 11.69 ± 2.99 | 0.680 | 1.21551 | 0.006 |
| lactate | HMDB00190 | 1.32(d), 4.11(q) | / | 216.39 ± 42.72 | 148.26 ± 38.06 | -0.657 | 1.2465 | 0.061 |
| threonine | HMDB00167 | 1.31(d), 3.58(d), 4.25(m) | + | 2.89 ± 1.19 | 6.20 ± 2.68 | 0.744 | 1.34323 | 0.001 |
| allantoin | HMDB00462 | 5.38(s) | + | 2.35 ± 1.44 | 5.73 ± 1.82 | 0.869 | 1.60685 | 0.001 |
| tyrosine | HMDB00158 | 6.89(m), 7.18(m) | / | 2.79 ± 3.49 | 5.16 ± 2.89 | 0.782 | 1.43234 | 0.067 |
| beatine | HMDB00235 | 3.25(s) | - | 165.64 ± 9.41 | 139.91 ± 27.77 | -0.529 | 1.15133 | 0.001 |
| N-phenylacetylglycine | HMDB00821 | 7.35(m), 7.41(m) | + | 0.30 ± 0.37 | 4.23 ± 0.35 | 0.873 | 1.63043 | 0.001 |
