# Supplementary material for: 1H NMR-Based Metabolomics Reveals the Antitumor Mechanisms of Triptolide in BALB/c Mice Bearing CT26 Tumors
Source: Front Pharmacol. 2019 Oct 11;10:1175. doi: 10.3389/fphar.2019.01175 (PMC6798008; doi:10.3389/fphar.2019.01175)
Supplement: Supplementary file 5 [file Table_2.docx]

| Metabolites | ID | Chemical shift | T vs M | Interal in Triptolide group ^a^  (mean ± std)×10^-2^ | Interal in Model group ^a^  (mean ± std)×10^-2^ | r ^b^ (T vs M)  (\|r\| >= 0.43) | VIP | p^c^ (Trip vs Model)  (p < 0.05) |
| --- | --- | --- | --- | --- | --- | --- | --- | --- |
| 2-Hydroxyisovalerate | HMDB00407 | 0.82(d), 0.95(d) | - | 1.45 ± 0.82 | 4.99 ± 1.70 | 0.783 | 2.0848 | 0.001 |
| 3-Hydroxybutyrate | HMDB00357 | 1.19(d), 2.30(m) | + | 28.05 ± 20.66 | 13.98 ± 3.96 | 0.613 | 1.05508 | 0.009 |
| valine | HMDB00883 | 0.98(d), 1.03(d) | + | 58.78 ±36.12 | 35.01 ± 19.20 | 0.732 | 1.03795 | 0.038 |
| isoleucine | HMDB00172 | 0.93(t), 0.99(d) | / | 26.64 ± 9.97 | 31.78 ± 7.73 | 0.738 | 1.04275 | 0.129 |
| 2-Hydroxyisobutyrate | HMDB00729 | 1.35(s) | - | 16.90 ± 4.07 | 60.73 ± 28.47 | -0.632 | 1.91583 | 0.001 |
| LDL/VLDL |  | 0.87(m), 1.27(m) | - | 12.97 ± 5.17 | 46.34 ± 17.42 | 0.678 | 2.02899 | 0.001 |
| unknown |  | 1.43(d) | / | 6.86 ± 2.43 | 6.58± 2.43 | 0.654 | 1.11462 | 0.709 |
| proline | HMDB00162 | 2.03(m), 2.36(m), 3.43(m) | - | 9.10 ± 2.93 | 11.36 ± 2.23 | 0.675 | 1.38476 | 0.025 |
| glycoprotein |  | 2.07(s) | - | 173.74 ± 43.69 | 214.73 ± 34.99 | -0.568 | 1.51384 | 0.009 |
| acetone | HMDB01659 | 2.22(s) | + | 10.14± 5.46 | 3.79 ± 1.64 | 0.555 | 1.45054 | 0.001 |
| pyruvate | HMDB00243 | 2.36(s) | + | 32.92 ± 4.30 | 23.95 ± 8.11 | -0.729 | 1.46004 | 0.002 |
| glutamine | HMDB00641 | 2.14(m), 2.46(m), 3.79(m) | + | 24.32 ± 1.80 | 16.27 ± 1.47 | 0.819 | 1.31441 | 0.004 |
| methionine | HMDB00696 | 2.13(s), 2.63(t) | + | 6.22 ± 3.91 | 7.0 ± 1.27 | 0.716 | 1.245 | 0.036 |
| trimethylamine | HMDB00906 | 2.89(s) | + | 5.55 ± 3.18 | 0.95 ± 0.97 | 0.594 | 1.75674 | 0.001 |
| creatine phosphate | HMDB01511 | 3.04(s), 3.93(s) | + | 52.84 ± 8.51 | 38.87 ± 12.35 | 0.93 | 1.50485 | 0.001 |
| taurine | HMDB00251 | 3.25(t), 3.42(t) | + | 51.54 ± 5.84 | 40.5 ± 8.07 | 0.858 | 1.68354 | 0.001 |
| glycerol | HMDB00131 | 3.55(m), 3.65(m) | - | 38.47 ± 7.47 | 55.14 ± 9.22 | -0.632 | 2.12286 | 0.001 |
| serine | HMDB00187 | 3.84(m), 3.96(m) | + | 17.79 ± 3.87 | 11.69 ± 2.99 | 0.812 | 1.63794 | 0.001 |
| lactate | HMDB00190 | 1.32(d), 4.11(q) | + | 96.32 ± 22.56 | 148.26 ± 22.09 | 0.191 | 1.32336 | 0.008 |
| allantoin | HMDB00462 | 5.38(s) | + | 7.40 ± 1.66 | 5.73 ± 1.82 | 0.908 | 1.10866 | 0.011 |
| tyrosine | HMDB00158 | 6.89(m), 7.18(m) | - | 3.47 ± 1.80 | 5.16 ± 0.47 | 0.817 | 1.8399 | 0.001 |
| N-phenylacetylglycine | HMDB00821 | 7.35(m), 7.41(m) | - | 5.41 ± 2.04 | 4.23 ± 1.17 | 0.826 | 1.05121 | 0.069 |
